# The Norwegian Adult Mental Health Registry for Quality Control in Specialized Mental Health Services: Protocol for a Nationwide Naturalistic Study

**DOI:** 10.2196/82696

**Published:** 2026-01-23

**Authors:** Tore Tjora, Kent Jensen, Ingrid Dieset, Katrine Kveli Fjukstad, Per Arne Holman, Martin Tesli, Inge Joa

**Affiliations:** 1 TIPS - Network for Clinical Research in Psychosis, Stavanger University Hospital, Gerd-Ragna Bloch Thorsens Gate 8, Stavanger, 4011, Norway, +47 51518000; 2 Department of Social Studies, Faculty of Social Sciences, University of Stavanger, Stavanger, Norway; 3 Universitetssykehuset i Nord Norge, Tromsø, Norway; 4 Division of Mental Health and Addiction, Oslo University Hospital, Oslo, Norway; 5 Department of Adult Psychiatry, Institute of Clinical Medicine, University of Oslo, Oslo, Norway; 6 Department of Psychiatry, Levanger Hospital, Nord-Trøndelag Hospital Trust, Levanger, Norway; 7 Department of Mental Health, Faculty of Medicine and Health Sciences, Norwegian University of Science and Technology, Trondheim, Norway; 8 Department of Patient Safety and Research, Lovisenberg Diakonale Sykehus AS, Oslo, Norway; 9 Division of Mental Health and Substance Abuse, Diakonhjemmet Hospital, Oslo, Norway; 10 Department of Mental Health, Norwegian Institute of Public Health, Oslo, Norway; 11 Faculty of Health, University of Stavanger, Stavanger, Norway

**Keywords:** mental illness, global burden of disease, national quality registry, adult patient, quality improvement, quality indicators, patient-reported outcome measures, data reuse

## Abstract

**Background:**

Mental disorders are highly prevalent, and they significantly impact individuals and society. Patients experiencing long-term, severe mental disorders with functional impairment and reduced quality of life often have a history of adolescent onset anxiety and depressive disorders. Despite the long-term costs to both patients and society, studies examining treatment effects over time and across diagnoses are scarce.

**Objective:**

The Norwegian Adult Mental Health Registry (NAMHR) aims to systematically reuse health data to monitor and improve treatment outcomes, patient safety, health service quality, and research. The registry addresses the need for comprehensive data on the effects and utility of mental health services, interventions, and therapy variants in specialized mental health care.

**Methods:**

The NAMHR is a nationwide naturalistic registry, including all Norwegian adults eligible for treatment in specialized mental health care services who have not opted out. Patients are automatically enrolled when treated in these services. The population includes patients treated in public specialized services and those treated in private services having a contract with public health services. The registry is based on secondary data from the Norwegian Patient Registry (NPR), patient-reported outcome measures (PROMs), patient-reported experience measures (PREMs), the Norwegian Registry for Primary Health Care (KPR), and several other sources, including electronic health records (EHRs). Data linkage uses unique national identity numbers, ensuring high-quality data. The registry collects information on diagnoses, treatments, medication, and patient-reported outcomes, providing a holistic approach to mental health care. Statistical analyses will be defined in each project.

**Results:**

As of December 2025, the NAMHR is approved and is being constructed. The registry anticipates enrolling up to 170,000 participants, with a new incidence rate of around 10,000 patients per year. Key predictors and outcomes include PROMs and PREMs, and automatically reported measures involving a wide range of data, including EHR data from inpatient and outpatient treatments, data from primary health care, data on job and education status, and data on cause of death. Enrollment is planned to start in 2026, initially by adding journal data and patient-reported data. Other sources will be included. The NAMHR has no planned end date. Results will be made available for internal quality improvement purposes, and data for research are expected to be available around mid-2026 for approved projects.

**Conclusions:**

The NAMHR will promote quality improvement initiatives and research, including registry-based randomized clinical trials. It will also be possible to link the NAMHR to a similar registry for children and adolescents, making it possible to follow patients from birth to death and supporting the monitoring of diagnostic drift. The NAMHR will inform health policy decisions at local, regional, national, and international levels, contributing to the evaluation and development of clinical guidelines and enhancing personalized treatment approaches.

## Introduction

Although mental disorders are highly prevalent and impact the lives of affected individuals and their next of kin, transdiagnostic nationwide quality-improvement programs are scarce. Two-thirds of mental disorders occur before the age of 25 years [1,2]. In both high-risk populations and the general population, mental disorders that develop in adolescence persist into adulthood, cause functional impairment, and reduce quality of life [3,4]. Mental disorders now account for at least 45% of the overall disease burden in those aged 10‐24 years [5,6] and represent a substantial burden at the societal level [7]. Early onset is associated with a longer duration of illness and additional comorbid disorders [8]. In particular, anxiety and depressive disorders rank high with regard to nonfatal health issues [9].

A quality registry is a systematic collection of data used to monitor and improve treatment outcomes, patient safety, and health service quality. In Norwegian national quality registers, the term “quality” especially refers to data accuracy, reliability, and relevance. To ensure a high level of data quality, the data must be complete, consistent, and up to date. A registry with good data, relevant measures, and relevant and accessible reports offers opportunities for policy makers, service managers, and researchers. Good quality also entails the active use of registries in health service improvement and innovation. The Norwegian Adult Mental Health Registry (NAMHR) will merge nationwide data sources, including patient-reported data, and is expected to provide new and unique data for both quality improvement and research. The data from the registry will contribute to the identification of variations in practice and the assessment of whether health services meet established quality criteria.

Established quality criteria include high effectiveness and safety, user involvement, resource coordination and utilization, high accessibility, and fair distribution. The NAMHR will provide clinicians and leaders with updated and accessible information on the effects and utility of health services, including specific interventions, therapy variants, and compulsory treatment use. This is important as specialized mental health care faces several challenges, and mental disorders are already the most expensive category of disorders in Norway [10,11].

The extent to which evidence-based clinical guidelines are implemented in daily clinical practice for specialized mental health care is unknown, and thus, it is difficult to monitor unwarranted variation [12]. Currently available data in Norway report on patient rates, admission rates, bed days, number of consultations, variation in staff specialities, rejected referrals, and waiting times. There is little information on patients’ perspectives. Although the volume, expertise, and availability of treatment are increasing, little is known about the effects of these increases on patients’ outcomes in the short and long term. There is a national need to follow patients’ pathways and progress; demonstrate and document treatment outcomes and effectiveness; perform quality control evaluations of treatment outcomes and effectiveness; and determine how treatments lead to changes in patients’ symptoms, functioning, and quality of life [13]. Furthermore, there is a need to uncover unwarranted variance across units, hospitals, and health regions. For example, a report from 2023 on attention-deficit/hyperactivity disorder (ADHD) diagnosis revealed large between-clinic variation in the incidence rate of ADHD [14].

Further adding to the importance of quality control is the evidence of increasing rates of mental disorders, including anxiety, depressive symptoms, psychological distress, self-harm, and suicide, among young people since early 2010 [15-21]. A recent Australian study of mental health and well-being (2020‐2022) revealed a 50% increase in mental disorders at the diagnosis level among people aged 16‐24 years since 2007, with an annual prevalence rate of 39% from 2020 to 2022 [16]. Similar but weaker tendencies are found in Norway, as reported in the SHOT Study (Students’ Health and Wellbeing Study) [22] and HUNT Study (Trøndelag Health Study) [23]. The presence of mental disorders among young people is a largely ignored risk factor for age-related medical illnesses [24]. The lifetime risk in a population that meets the criteria for a diagnosable mental disorder is estimated to be 50% [25]. If these disorders are not treated, they can become major causes of reduced quality of life, high costs to the family, and high disability-adjusted life years [26]. Suicide remains the fourth most common cause of death among people aged 15‐29 years globally [5], and mental disorders are some of the most frequent reasons for visits to general practitioners. Patients with mental disorders have 5‐15 life-years lost (LYL) [27], and patients with serious mental illnesses, such as schizophrenia, have 9‐12 LYL [28]. The most common causes of death overall are natural causes, such as cardiovascular diseases and cancer, many of which are preventable and treatable [29].

**Figure 1.**
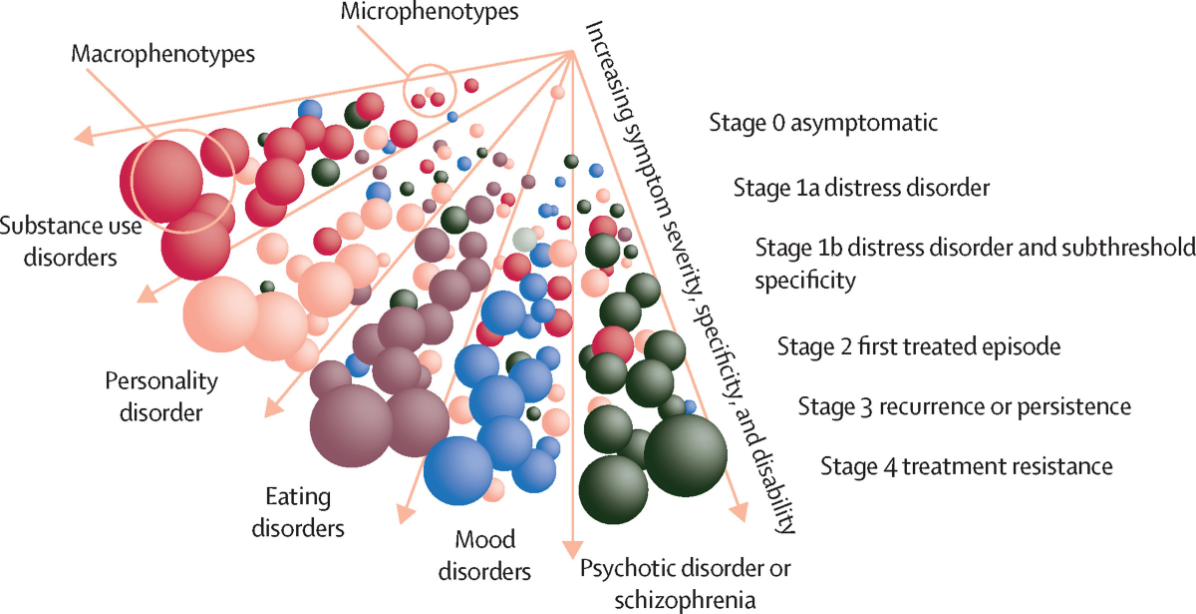
Heuristic clinical staging model for mental disorders (reproduced with permission from McGorry and Mei [36]).

In real-life clinical psychiatric settings, it is relatively common for diagnoses to change over time. An individual receiving an anxiety diagnosis in early adolescence might be assessed as having depression in late adolescence before being diagnosed with schizophrenia in their mid-20s. Adding to this, the diagnostic criteria themselves have changed, including the *Diagnostic and Statistical Manual of Mental Disorders* (*DSM*) [30] and the *International Statistical Classification of Diseases and Related Health Problems* (*ICD*) [31]. The individual diagnostic “drift” and criteria “drift” highlight the importance of transdiagnostic quality registers. Diagnostic drift also highlights the importance of incorporating new models or frameworks in specialized mental health quality control, and a suitable model is clinical staging. The clinical staging model [32-36] (Figure 1) presents a new and important longitudinal perspective. Clinical staging is congruent and synergistic with many developmental and related conceptual frameworks and allows for exploring mechanisms underpinning the onset and course of mental disorders. The clinical staging model places people along a multidimensional continuum from health to illness in order to capture elements of risk, onset, course, and prognosis. This transdiagnostic model [32,36-38] recognizes the fluid nature of mental disorders and distinguishes between early clinical stages (with low rates of progression to more severe disorders) and later stages (with higher rates of persistence and impairment).

The model illustrates clusters of early symptoms (microphenotypes) and their potential progression into clear and often comorbid syndromes (macrophenotypes). This progression across stages is characterized by increasing symptom severity, specificity, and disability (represented by the sphere size; the spheres and colors represent phenotypes).

The clinical staging model is suitable as an overarching framework for the NAMHR to guide the ongoing selection of unwarranted variations to monitor. Currently, even established diagnostic-specific Norwegian care pathways [39] lack implementation studies. These care pathways specify time frames for assessment, treatment, and clinical evaluation and are based on previous findings that show positive prognostic effects of early diagnosis and treatment [40]. In line with clinical staging, the purpose of care pathways is to improve services across functions, activities, and units, as well as the wider health and welfare service system. The care pathway packages follow the same principles as those previously implemented for cancer [41]. Collecting data on care pathways enables the NAMHR to observe continuous chains of treatment and follow-up across service providers and to provide data on unnecessary delays in assessment, treatment, care, and follow-up in order to contribute to equal quality of care across geographical regions. Findings from several care pathways may provide data for a more overarching model like the clinical staging model. For example, poor coordination of medical health care has been associated with early mortality and a lack of physical health checks, prescriptions, and medical procedures [42-44]. More importantly, it is unknown whether and to what extent care pathways improve mental, medical, and functional outcomes. Additionally, there are no current national quality indicators that measure patients’ mental health treatment outcomes, and such indicators are not linked to the degree of compliance with care pathways.

Patient-reported measures are important in health care in general, but patients’ own experiences are especially important in mental health care as mental disorders manifest as challenges and disturbances in the experience of oneself and the world. In research settings, standardized and validated self-report measures often define outcomes. In mental health care, other outcomes, such as diagnoses and waiting times, are more frequent. The systematic collection of patient-reported outcome measures (PROMs) [45] and patient-reported experience measures (PREMs) [46,47] is increasing. The PROMs subgroup of routine outcome monitoring (ROM) involves clinical data primarily intended as part of a medical journal for individual documentation and treatment. Great Britain’s public health services have used such ROM data to document treatment effects [48,49] at the individual level. However, aggregated ROM data may be used as process measures in quality registries to detect unwarranted variations. Overall, the patient perspective is emphasized as an important aspect of quality in health services. To enhance the patient perspective, the National Mental Health Service Organization (Mental Helse Norge [50]) participated in the development of the NAMHR and has 2 members on the advisory board. The patient perspective includes the patient’s satisfaction with treatment and provides subjective measures of effect and change. In mental health care, several PROMs are at a level of precision to be accepted as outcome variables in a quality registry [48], but are not sufficiently used at the system level. In Norway, this need is highlighted in the government’s (Stortinget’s) report on quality and patient safety [51].

The NAMHR’s purpose is to provide a platform for transdiagnostic, nationwide quality improvement. One such issue is increased knowledge of “personalized treatment” in adults (eg, what works, when, where, in what way, and for whom). This is primarily fulfilled by facilitating the statutory duty for quality improvement that specialist health care providers have, and secondarily by delivering data for research. Specialized mental health care services in Norway are divided into child and adolescent services and adult services, resulting in 2 separate registers. Given the abovementioned importance of early interventions and longitudinal perspectives, this is suboptimal. However, as both registers have a common system architecture, ethically approved research studies may use merged data, following individuals from birth to death.

## Methods

### Study Design

The sample will include individuals who have been referred to specialized mental health care services and have not opted out. As individuals may be included at any time and included individuals may file reservations (opt out) at any time, both the population size and sample size will vary. The total population was approximately 170,000 participants as of 2023 [52]. The population includes patients treated at public specialized mental health care services and similar private services having a contract with public health services (contract specialists). Mental health services funded outside public health services (eg, privately or through insurance) are not considered. According to the South-Eastern Norway Regional Health Authority (the largest of the Norwegian health authorities), only 1% of licensed health personnel were working in private specialized health services in 2025 [53], which indicates that the vast majority of specialized health care services are funded publicly in Norway. According to the Norwegian Patient Registry (NPR) [54], the yearly treated incidence rate is estimated at approximately 10,000 patients. The start of data collection has been planned for autumn 2025. Patients treated prior to the start will be considered as new cases, resulting in an initial overestimation of incidence rates. The degree of registry coverage is defined as the number of nonreserved participants divided by the number of eligible participants. The coverage is currently unknown as neither data collection nor reservation has started. The NAMHR anticipates similar coverage as that of the reservation-based Norwegian Diabetes Register for Adults, which reported a nationwide coverage of 88% in 2023 [55].

The NAMHR was approved in late 2023 and is designed as a national naturalistic follow-along study based on all Norwegian adults eligible for treatment in specialized mental health care services, who have not opted out of participation (detailed inclusion and exclusion criteria are provided below). This protocol is compliant with the standards outlined in the SPIRIT (Standard Protocol Items: Recommendations for Interventional Trials) 2013 Statement [56]. The SPIRIT checklist is provided in Checklist 1. The trial is registered at ClinicalTrials.gov (NCT06115200) [57,58].

### Patient and Public Involvement

The NAMHR is run by Stavanger University Hospital and managed by an advisory board. The advisory board consists of clinicians and researchers from all 4 regional health trust organizations in Norway, representatives from work organizations (psychiatrists, psychologists, and nurses), and representatives from large Norwegian organizations for users and their relatives. The user (patient) representatives have been actively involved from the start and have contributed to many areas, especially quality topic selection and prioritization. The interests of the public are maintained through representatives from the 4 regional health trusts.

### Population

#### Inclusion Criteria

All individuals referred for treatment to specialized mental health care services, who have not already opted out, will be included. Patients will be informed about the registry as part of the general information received by all patients with the right to undergo treatment at specialized mental health care services. To allow patients time to opt out, data from new eligible patients will not be used for the first 30 days after inclusion. If new eligible patients do not opt out within 30 days, their data, including data from the first 30 days, will be included in the registry.

#### Exclusion Criteria

##### Patient Reservation

Patients who opt out (reserve themselves) will be excluded. Patients may remove or reserve themselves at any time, including prior to any contact with specialized mental health services and possible registry inclusion. Patients may remove their reservation at any time. Patients who remove their reservation will be included in the registry again; however, data from the reservation period will not be regenerated.

##### Patients Already Included in Other Registries

Patients between 18 and 21 years of age who are already included in the KVABUP registry (Child and Adolescent Mental Health Registry) will be excluded. Moreover, patients older than 65 years who are included in the KVALAP registry (Quality Register for Old Age Psychiatry Patients) will be excluded.

### Recruitment and Follow-Up Procedures

As the registry is based on the secondary use of data, participants will be recruited and followed up as part of their treatment. Treatment will not be affected in any way by inclusion or exclusion in the registry, and the registry will not initiate contact or follow-up, with 2 exceptions. First, if the registry discovers data that are clearly incorrect (eg, a height of more than 3 meters), the registry will contact the data owner for correction. Second, the registry will follow up with patients who contact the registry directly. The registry has established procedures to handle requests for correcting, deleting, and viewing data of individual participants and has introduced procedures for patients to remove themselves or other patients under their care from inclusion.

### Data Sources

**Figure 2.**
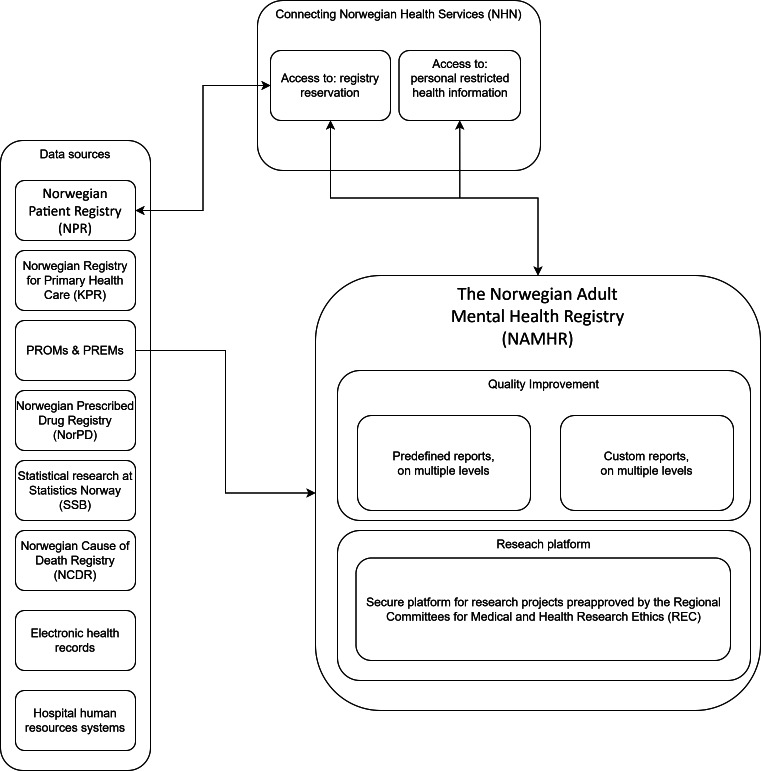
Norwegian Adult Mental Health Registry data flow diagram. PREMs: patient-reported experience measures; PROMs: patient-reported outcome measures.

The NAMHR uses previously collected data (secondary use) from mental health care services and combines data from other sources, such as government records and registries (Figure 2). The main information source for the NAMHR is the NPR [54]. The NPR contains comprehensive information about individuals referred to or treated by specialized health care providers like hospitals, outpatient clinics, and contract specialists. The NPR includes personal details (eg, age, sex, and address), claim data (eg, provider information, episodes of care, patient rights, time of death, and registry consent), medical information (eg, medical specialty, diagnoses, procedures, timestamps on episodes of care, compulsory treatment, and substance use), and social information (eg, housing conditions and care situations). The data in the NPR stem from mandatory monthly reports from all the public health trusts, private and nonprofit organizations under the public health trusts (contract), and private contract specialists [59]. The reports are automatically generated from the electronic health records (EHRs) and reflect the interdisciplinary collaboration among medical doctors, psychologists, nurses, and individuals from other disciplines entering data in the EHRs.

The NAMHR will enrich the NPR data by merging multiple other sources. To enhance patient perspectives and experiences, PROMs and PREMs are included. PROMs and PREMs are currently collected for individual treatments. The NAMHR plans to integrate all platforms of PROMs and PREMs used in Norway.

The NAMHR will also merge data from hospital EHRs and data from the Norwegian Registry for Primary Health Care (KPR) [60]. The latter provides information on the direct and indirect contact of general practitioners with patients. Further, the NAMHR has been authorized to merge other sources (details are provided in Figure 2).

As the NAMHR is primarily a tool for quality improvement in specialized mental health care in Norway, both exposure and outcome variables may change as the need for quality indicators changes over time. The list of exposure and outcome variables should be considered tentative.

### Outcomes: Quality Indicators

The purpose of the national quality indicator system [61] is to ensure equal access to good-quality health services for the population based on the framework of the Health Care Quality and Outcomes program of the Organisation for Economic Co-operation and Development [62-64]. The national quality indicators serve several purposes: (1) they provide central health authorities with a basis for monitoring, prioritization, and management; (2) they provide managers at all levels with a basis for quality improvement; and (3) they contribute to public transparency on quality and variation in services.

The NAMHR’s quality indicators are dynamic, as they aim to reflect quality challenges in mental health care for adults at any given time. The register’s proposed quality indicators (Table 1) are based on and comply with recommendations in national guidelines. As of 2025, in Norway, there are five national guidelines in the field of mental health (adults): (1) National professional guidelines for ADHD/hyperkinetic disorder [65]; (2) National professional guidelines for the prevention of suicide in mental health care [66]; (3) National professional guidelines for the early detection, assessment, and treatment of eating disorders [67]; (4) National professional guidelines on the use of electroconvulsive therapy [68]; and (5) Guidelines on psychotic disorders and medications [69].

**Table 1.** Initial Norwegian Adult Mental Health Registry quality indicators.

Overarching theme and quality indicator (measure)		Type	Expected level for the measure
Examination and diagnostics			
Proportion of incident (new) patients diagnosed with structured diagnostic instruments (eg, SCID-CV^a^ and MINI^b^ 7.0.2)		Process	≥90%
Somatic health and cardiovascular risk			
Proportion of incident (new) patients whose BMI is measured		Process	≥90%
Proportion of incident (new) patients whose lipid and HbA_1c_ levels are measured		Process and results	≥90%
Proportion of incident (new) patients whose blood pressure is measured		Process and results	≥90%
Outcome/results			
Proportion of patients who use the mental health feedback tool for adults		Process and results	≥90%
Similar results for comparable groups (CORE-OM^c^) [70]		Results	Group difference in improvement >5 points
Improved quality of life (WHODAS^d^) [71]		Results	Share of participants who report improved quality of life

a SCID-CV: Structured Clinical Interview for *DSM-5*, Clinician Version.

b MINI: Mini International Neuropsychiatric Interview.

c CORE-OM: Clinical Outcomes in Routine Evaluation – Outcome Measure.

d WHODAS: World Health Organization Disability Assessment Schedule.

### Measures

#### Individual and Structural Variables (Predictors/Covariates)

Individual variables reported at baseline include age, gender, tentative diagnosis, and reason for referral. Structural variables consist of system-level variables such as the name of the institution, health region, and county. System-level variables collected from hospital record systems include staff size, personnel turnover, tenure, the percentage of doctors who are psychiatrists, the percentage of psychologists with relevant specializations, and the number of available hospital rooms. The list of variables is tentative, may vary by health region, and may change over time.

#### Process Variables (Mediators/Moderators)

Data on processes will be collected at the individual and system levels. Individual-level data include the type and length of treatment, and structured data from EHRs, including BMI, blood pressure, and the type and dosage of medication. Additionally, PREMs and PROMs will be collected. Examples are detailed in Textbox 1. The process variables also include time-variant variables at the system level. For example, case-load indicators may be defined as the number of consultations registered divided by the number of employees.

Textbox 1. **Example structural, process, and outcome variables in the Norwegian Adult Mental Health Registry.**

Predictor variables (independent); individual and structural levels

- Symptoms and function at baseline; patient-reported outcome measures (PROMs) (Clinical Outcomes in Routine Evaluation–Outcome Measure [CORE-OM], Alcohol Use Disorders Identification Test [AUDIT] [72], and Drug Use Disorders Identification Test [DUDIT] [73])
- Age (Norwegian Patient Registry [NPR]) and gender (NPR)
- Referral date (NPR)
- Assessment date (NPR)
- Start date, both outpatient and inpatient (NPR)
- Unit name and description (NPR)
- Prior referral/treatment (NPR)
- Prior episode of compulsory mental health care (NPR)
- Suicide assessment (NPR)
- System-level variables, such as turnover, tenure, percentage of doctors who are psychiatrists, percentage of psychologists with relevant specializations, and number of available hospital rooms

Process variables; individual level

- Symptoms and function at multiple times during treatment; PROMs (CORE-OM, AUDIT, and DUDIT)
- Hours spent on outpatient treatment
- End date (NPR)
- Duration of treatment (NPR)
- Use of structured symptom and function assessment instruments (NPR)
- Structured codes for diagnoses and procedures from the national patient registry (NPR)
- Quality of life assessment (World Health Organization Disability Assessment Schedule [WHODAS])
- Ongoing compulsory mental health care (NPR)
- Suicide assessment
- Type of given treatment, standardized procedure codes (NPR)
- Somatic: blood pressure, height, weight, and other relevant tests (electronic health record [EHR])
- Medication: type, administration, dosage (Norwegian Prescribed Drug Registry [NorPD])
- System-level variables, such as turnover, tenure, percentage of doctors who are psychiatrists, percentage of psychologists with relevant specializations, and number of available hospital rooms

Result variables (outcome); individual and structural levels

- Symptoms and function at discharge; PROMs (CORE-OM, AUDIT, and DUDIT)
- PROMs, quality of life change (WHODAS)
- Patient-reported experience measures; overall satisfaction with mental health care (Pasient Opplevelser - patients’ satisfaction with treatment [PASopp])
- Mortality rate and cause of death (Norwegian Cause of Death Registry [NCDR])
- Outcome measures regarding primary care follow-up, such as individual treatment plan, general practitioner assessment, municipality coordinator, type of work, and ongoing education (Norwegian Registry for Primary Health Care [KPR])
- Compulsory mental care as part of aftercare (NPR)
- Suicide assessment
- Medication type, administration, and dosage in epicrisis on discharge (EHR and NorPD)

#### Outcome Variables

PROMs are registered at baseline, multiple times during the intervention, at postintervention, at discharge, and 6 months after discharge. The PROM battery may vary across health regions, and a reliable change index score [74] may be used for comparison. Outcome variables at discharge include discharge diagnosis, treatment type, treatment length, treatment frequency, region ID, and facility ID. The list of outcome variables is tentative, may vary from one health region to another, and may change over time. However, the abovementioned variables are expected to be stable over time and place.

### Statistical Analysis

Statistical analyses will vary from project to project, and for both internal quality improvement projects and published research papers, a wide range of statistical approaches may be used. “Statistical process control” [75] may be used, as it allows close monitoring of longitudinal changes in complex systems. Classic approaches, ranging from descriptive cross-sectional studies (case-control and cohort studies) to randomized controlled trials (RCTs) based on registry data may also be performed. Further, the vast amount of data, especially many predictors from different sources, may suggest a more multilevel approach. For example, exploratory factor analysis may be used to explore factor structures, which, in combination with structural equation modeling, may be used to explore relationships between observed and unobserved (latent) variables. Latent class models and latent growth models [76] may be used to explore the difference in developmental trajectories. Exploratory studies and quality improvement projects may use forecast models based on time-series data from the registry, both to describe ongoing trends and plan the future of health care. The registry may also form the basis for machine learning approaches, such as neural networks, to both model very complex, longitudinal data in an understandable way and establish models for more precise prediction of mental disorders.

### Future Registry Expansion

The registry has been approved for the inclusion of data from other national health-related registries, such as the Norwegian Prescribed Drug Registry (NorPD) [77], the Norwegian Cause of Death Registry (NCDR) [78], and the KPR [60]. The NorPD includes data on drugs prescribed by doctors in primary health care and drugs prescribed and administered as part of treatment in specialized health care. The NAMHR has also been approved for the inclusion of data from other national general registries, such as income, tax, and social benefits.

### Data Linkage and Storage

The data will come from multiple sources (detailed above) and will be linked using the unique national identity number. Each patient will be given a random number for internal use. The data will be stored within the same infrastructure as most of the data sources that are included, owned, and run by Connecting Norwegian Health Services (Norsk helsenett) [79].

Risk and vulnerability assessments and data protection impact assessments [80] have been performed to ensure compliance with the General Data Protection Regulation (GDPR) [81] and will be revised in accordance with procedures in Helse Vest Health Care Trust.

### Trial Duration

The registry has no planned end date.

### Ethical Considerations

In Norway, legislation and regulation regarding quality improvement and research are handled separately. The registry is primarily part of the ongoing quality improvement program imposed by the Norwegian law on specialized health care, section 3.4a [82]. All national medical quality registries are appointed by the Norwegian Directorate of Health. The registry will be run by a board, as detailed earlier in the *Methods* section. Data collection and usage for quality improvement are regulated by the abovementioned laws and according to GDPR regulations and standards [83].

The participants will not receive compensation for any inconvenience or time spent. The NAMHR will reuse health data, and the participants do not have to take any additional effort. The participants have the right to opt out (reserve themselves), correct their data, and delete their data. The NAMHR will follow the European Union’s data privacy law (GDPR), which will ensure privacy and confidentiality.

The data will be made available for use in research. In Norway, “all medical and health research projects must be preapproved by the *Regional Committees for Medical and Health Research Ethics*” [84]. Ethical approval must be obtained before the beginning of the project and data delivery from the NAMHR. The full study protocol and relevant statistical code may be provided on request.

The results of the research projects will be published in peer-reviewed journals and at research conferences. Authorship will follow the “Vancouver Recommendations.” Open-access journals will be preferred. Important protocol modifications will be reported at ClinicalTrials.gov [57].

## Results

The NAMHR was approved as a mandatory quality instrument in late 2023. As of December 2025, the NAMHR is approved and is being constructed. The study anticipates enrolling up to 170,000 participants, with a new incidence rate of around 10,000 patients per year. Key predictors and outcomes include both PROMs and PREMs, as well as automatically reported measures on a wide range of data, including EHR data from inpatient and outpatient treatments, data from primary health care, data on job and education status, and data on cause of death. The NAMHR has no planned end date. Data for both quality improvement and research projects are planned to be available from mid-2026.

## Discussion

### Overview

This paper outlines our study protocol for the NAMHR. The primary goal of the NAMHR is to monitor and develop future mental health care, and it will provide important knowledge that benefits all patients and the general population. The NAMHR provides a unique opportunity by gathering large-scale, high-quality, and representative data. As Norwegians have free access to public health insurance, the vast majority of Norwegians use public health care, resulting in highly representative data. Further, public-delivered or commissioned services require public and private providers to deliver standardized claim data to the NPR.

### Hypothesized Main Findings

The registry may be used to investigate the effects of various treatments and as a nationwide control for RCTs. The NAMHR combines data from all major EHRs in Norway, ranging from data on medication and medication administration from general practitioners to high-temporal-resolution drug monitoring systems for inpatient treatment. Hence, the NAMHR will be one of the first nationwide data sources for monitoring longitudinal medication usage for individual patients and combining in- and outpatient prescriptions and psychological treatments provided. This may be done in combination with service provision variables and PROMs or PREMs, hence providing a unique opportunity for quality improvement. In addition, it will provide the opportunity to perform large-scale research on the long-term effects of psychological and drug treatments. The NAMHR will also be a platform for registry-based randomized clinical trials, which are prospective randomized trials that use a clinical registry for one or several major functions to conduct trials and report outcomes. The advisory board has already decided that involuntary treatment in mental health services is one of the first quality indicators that will be targeted for quality improvement and research.

### Comparison to Prior Work

In contrast to the other Nordic mental health registries, the NAMHR is organized as a transdiagnostic mental health registry, which makes the NAMHR more suitable for investigating the fluid nature of diagnostics across the lifespan [38]. Further, it provides an opportunity to detect and end mental health interventions with low returns on investment [85]. The combination of a transdiagnostic approach and a large sample size provides a possibility of monitoring compliance with a wide range of clinical guidelines. The registry also represents an opportunity to inform new clinical guidelines, including guidelines involving more than one diagnostic category, a possibility few mental health registries possess. When unwarranted variation is revealed, the advisory board and each hospital trust may use this information to develop new quality indicators. These indicators and the assessment of their effects on unwarranted variation may result in new clinical guidelines based on large-scale empirical data. This is not new; however, automated data collection and merging may result in a much cheaper and faster process. When new guidelines are introduced, the system for monitoring guideline implementation and compliance is already up and running. Traditionally, discharged patients who were not seen again were assumed to have had a good course of treatment. However, the use of more objective data, such as sick leave and taxes paid, which are included in the NAMHR, can provide more accurate information.

Hence, data delivered from the NAMHR will be highly relevant for health policy decision-making at the local, regional, national, and international levels. For example, care pathway packages are implemented with limited evidence of their efficacy or effectiveness in mental health. Thus, it is relevant for policymakers to evaluate the effects on treatment delivery and outcomes. The wide variation in health service delivery is a cause for concern among policymakers at all levels [14], but traditional RCTs fall short of defining the best practices. Some important questions are as follows: Can high rates of involuntary treatment be justified by reduced suicide and violence rates, or does this reflect the unnecessary use of coercive measures? Do early intervention policies produce improved outcomes for all patients in the system or only for those selected for early intervention at the expense of other patients [86]? Can patient-generated data with personalized psychometrics enhance treatment processes, shared decision-making, and outcomes?

### Strengths and Limitations

The NAMHR provides a relatively high temporal resolution with a longitudinal perspective and may be used to describe various trajectories or typical pathways through health services. In addition, the registry may be used to calculate which of the abovementioned trajectories are associated with preferable outcomes, ranging from subjective outcomes, such as PREMs and PROMs, to more objective outcomes, such as work status and taxes paid. The registry will be linked to the upcoming national quality registry for child and adolescent specialized mental health services for research purposes after the development of trajectories. As mentioned, the NAMHR is organized as a transdiagnostic mental health registry, which is also considered a strength.

The reuse of data is one of the greatest strengths of the NAMHR, but it can also be considered a major limitation. As the registry only reuses data collected for other purposes, the registry has no direct influence on what is collected. Another possible limitation is the unknown degree of reservation. If the percentage of patients who chose to reserve themselves (opt out) from the registry is large and/or associated with key predictors or outcomes, it will affect to what degree the findings are generalizable to other populations.

### Dissemination Plan

Findings from the registry will have 2 main outlets. First, quality improvements will be monitored and reported through a dashboard available for health services and reports. Second, as mentioned above, data will be made available for projects with ethical approval from the Regional Committees for Medical and Health Research Ethics (REC) [84]. The NAMHR does not decide the publication channel of ethically approved research projects. However, a proper plan for dissemination is necessary for each project to obtain ethical approval from the REC. The NAMHR will strongly suggest using open-access journals. The NAMHR will publish new papers on ClinicalTrials.gov [57].

### Conclusion

The NAMHR, in addition to other Nordic mental health registries and similar registries in other countries, may play an important role in psychiatric health care worldwide. The registry will deliver data for both quality improvement and research with real-world outcomes, including patient symptoms, functioning, and quality of life.

## Supplementary material

10.2196/82696 Checklist 1 SPIRIT checklist.

## Abbreviations

ADHD: attention-deficit/hyperactivity disorder
*DSM*: *Diagnostic and Statistical Manual of Mental Disorders*
EHR: electronic health record
GDPR: General Data Protection Regulation
*ICD*: *International Statistical Classification of Diseases and Related Health Problems*
KPR: Norwegian Registry for Primary Health Care
LYL: life-years lost
NAMHR: Norwegian Adult Mental Health Registry
NCDR: Norwegian Cause of Death Registry
NorPD: Norwegian Prescribed Drug Registry
NPR: Norwegian Patient Registry
PREM: patient-reported experience measure
PROM: patient-reported outcome measure
RCT: randomized controlled trial
REC: Regional Committees for Medical and Health Research Ethics
ROM: routine outcome monitoring
SPIRIT: Standard Protocol Items: Recommendations for Interventional Trials

## References

[1] Kessler RC, Amminger GP, Aguilar-Gaxiola S, Alonso J, Lee S, Ustün TB (2007). Age of onset of mental disorders: a review of recent literature. Curr Opin Psychiatry.

[2] Solmi M, Radua J, Olivola M (2022). Age at onset of mental disorders worldwide: large-scale meta-analysis of 192 epidemiological studies. Mol Psychiatry.

[3] Mulraney M, Coghill D, Bishop C (2021). A systematic review of the persistence of childhood mental health problems into adulthood. Neuroscience & Biobehavioral Reviews.

[4] Sivertsen B, O’Connor RC, Nilsen SA (2024). Mental health problems and suicidal behavior from adolescence to young adulthood in college: linking two population-based studies. Eur Child Adolesc Psychiatry.

[5] Suicide. World Health Organization.

[6] Patton GC, Sawyer SM, Santelli JS (2016). Our future: a Lancet commission on adolescent health and wellbeing. The Lancet.

[7] Christensen MK, Lim CCW, Saha S (2020). The cost of mental disorders: a systematic review. Epidemiol Psychiatr Sci.

[8] Caspi A, Houts RM, Ambler A (2020). Longitudinal assessment of mental health disorders and comorbidities across 4 decades among participants in the Dunedin Birth Cohort Study. JAMA Netw Open.

[9] Tollånes MC, Knudsen AK, Vollset SE, Kinge JM, Skirbekk V, Øverland S (2018). Disease burden in Norway in 2016. Tidsskr Nor Laegeforen.

[10] Kinge JM, Dieleman JL, Karlstad Ø (2023). Disease-specific health spending by age, sex, and type of care in Norway: a national health registry study. BMC Med.

[11] Kinge JM, Sælensminde K, Dieleman J, Vollset SE, Norheim OF (2017). Economic losses and burden of disease by medical conditions in Norway. Health Policy.

[12] Sutherland K, Levesque JF (2020). Unwarranted clinical variation in health care: definitions and proposal of an analytic framework. J Eval Clin Pract.

[13] (2021). Riksrevisjonens undersøkelse av psykiske helsetjenester [Article in Norwegian]. https://www.stortinget.no/no/Saker-og-publikasjoner/Publikasjoner/Dokumentserien/2020-2021/dok3-202021/dok3-202021-013/.

[14] Widding-Havneraas T, Markussen S, Elwert F (2023). Geographical variation in ADHD: do diagnoses reflect symptom levels?. Eur Child Adolesc Psychiatry.

[15] Office of the Surgeon General (OSG) (2021). Protecting Youth Mental Health: The US Surgeon General’S Advisory.

[16] (2023). National study of mental health and wellbeing: 2020-2022. Australian Bureau of Statistics.

[17] Jensen HAR, Davidsen M, Møller SR, Román JEI, Kragelund K (2022). Danskernes Sundhed – Den Nationale Sundhedsprofl 2021 [Article in Danish].

[18] Newlove-Delgado T, Marcheselli F, Williams T, Mandalia D, Davis J, McManus S (2022). Mental health of children and young people in England 2022. https://openaccess.city.ac.uk/id/eprint/30558/1/Mental%20Health%20of%20Children%20and%20Young%20People%20in%20England%202022%20-%20wave%203%20follow%20up%20to%20the%202017%20survey%20-%20NDRS.pdf.

[19] Twenge JM, Joiner TE, Rogers ML, Martin GN (2018). Increases in depressive symptoms, suicide-related outcomes, and suicide rates among U.S. adolescents after 2010 and links to increased new media screen time. Clin Psychol Sci.

[20] Naghavi M, Global Burden of Disease Self-Harm Collaborators (2019). Global, regional, and national burden of suicide mortality 1990 to 2016: systematic analysis for the Global Burden of Disease Study 2016. BMJ.

[21] (2022). Mental health of children and young people in England 2022 - wave 3 follow up to the 2017 survey. https://digital.nhs.uk/data-and-information/publications/statistical/mental-health-of-children-and-young-people-in-england/2022-follow-up-to-the-2017-survey.

[22] Sivertsen B, Knudsen AKS, Kirkøen B (2023). Prevalence of mental disorders among Norwegian college and university students: a population-based cross-sectional analysis. Lancet Reg Health Eur.

[23] Krokstad S, Weiss DA, Krokstad MA (2022). Divergent decennial trends in mental health according to age reveal poorer mental health for young people: repeated cross-sectional population-based surveys from the HUNT Study, Norway. BMJ Open.

[24] Moffitt TE, Caspi A (2019). Psychiatry’s opportunity to prevent the rising burden of age-related disease. JAMA Psychiatry.

[25] Mykletun A, Knudsen AK, Mathiesen KS (2009). Psykiske lidelser i Norge: Et folkehelseperspektiv [Article in Norwegian]. https://www.fhi.no/publ/eldre/psykiske-lidelser-i-norge-et-folkeh/.

[26] Arias D, Saxena S, Verguet S (2022). Quantifying the global burden of mental disorders and their economic value. EClinicalMedicine.

[27] Plana-Ripoll O, Pedersen CB, Agerbo E (2019). A comprehensive analysis of mortality-related health metrics associated with mental disorders: a nationwide, register-based cohort study. The Lancet.

[28] Tesli M, Degerud E, Plana-Ripoll O (2022). Educational attainment and mortality in schizophrenia. Acta Psychiatr Scand.

[29] Gronholm PC, Chowdhary N, Barbui C (2021). Prevention and management of physical health conditions in adults with severe mental disorders: WHO recommendations. Int J Ment Health Syst.

[30] American Psychiatric Association (2013). Diagnostic and Statistical Manual of Mental Disorders, Fifth Edition.

[31] (2019). International Statistical Classification of Diseases and Related Health Problems 10th Revision. World Health Organization.

[32] McGorry P, Nelson B (2016). Why we need a transdiagnostic staging approach to emerging psychopathology, early diagnosis, and treatment. JAMA Psychiatry.

[33] Iorfino F, Scott EM, Carpenter JS (2019). Clinical stage transitions in persons aged 12 to 25 years presenting to early intervention mental health services with anxiety, mood, and psychotic disorders. JAMA Psychiatry.

[34] McGorry P, van Os J (2013). Redeeming diagnosis in psychiatry: timing versus specificity. Lancet.

[35] McGorry PD, Hickie IB, Yung AR, Pantelis C, Jackson HJ (2006). Clinical staging of psychiatric disorders: a heuristic framework for choosing earlier, safer and more effective interventions. Aust N Z J Psychiatry.

[36] McGorry PD, Mei C (2021). Clinical staging for youth mental disorders: progress in reforming diagnosis and clinical care. Annu Rev Dev Psychol.

[37] McGorry PD, Hartmann JA, Spooner R, Nelson B (2018). Beyond the “at risk mental state” concept: transitioning to transdiagnostic psychiatry. World Psychiatry.

[38] McGorry PD, Mei C, Dalal N (2024). The Lancet Psychiatry Commission on youth mental health. Lancet Psychiatry.

[39] Ådnanes M, Høiseth JR, Magnussen M, Thaulow K, Kaspersen SL (2021). Pakkeforløp for psykisk helse og rus – brukere, pårørende og fagfolks erfaringer [Article in Norwegian]. https://www.sintef.no/publikasjoner/publikasjon/1961108/.

[40] (2021). Forløpstid for utredning i psykisk helsevern for voksne [Article in Norwegian]. Helsedirektoratet.

[41] Pakkeforløp for kreft (treatment packages for cancer) [Article in Norwegian]. Helsedirektoratet.

[42] Haussleiter I, Emons B, Hoffmann K, Juckel G (2021). The somatic care situation of people with mental illness. Health Sci Rep.

[43] Prior A, Vestergaard CH, Vedsted P (2023). Healthcare fragmentation, multimorbidity, potentially inappropriate medication, and mortality: a Danish nationwide cohort study. BMC Med.

[44] Rodgers M, Dalton J, Harden M, Street A, Parker G, Eastwood A (2018). Integrated care to address the physical health needs of people with severe mental illness: a mapping review of the recent evidence on barriers, facilitators and evaluations. Int J Integr Care.

[45] Patient-reported outcome measures (PROMs). Canadian Institute for Health Information.

[46] Fernandes S, Fond G, Zendjidjian XY (2020). Measuring the patient experience of mental health care: a systematic and critical review of patient-reported experience measures. Patient Prefer Adherence.

[47] Olsen RV, Garratt AM, Iversen HH, Bjertnaes OA (2010). Rasch analysis of the Psychiatric Out-Patient Experiences Questionnaire (POPEQ). BMC Health Serv Res.

[48] Gelkopf M, Mazor Y, Roe D (2022). A systematic review of patient-reported outcome measurement (PROM) and provider assessment in mental health: goals, implementation, setting, measurement characteristics and barriers. Int J Qual Health Care.

[49] Barkham M, De Jong K, Delgadillo J, Lutz W (2023). Routine outcome monitoring (ROM) and feedback: research review and recommendations. Psychother Res.

[50] Mental Helse Norge.

[51] (2016). Kvalitet og pasientsikkerhet 2016 [Article in Norwegian]. https://www.regjeringen.no/no/dokumenter/meld.-st.-6-20172018/id2581316/.

[52] (2023). Aktivitetsdata for spesialisthelsetjenesten [Article in Norwegian]. https://www.fhi.no/globalassets/dokumenterfiler/rapporter/2024/aktivitet-phv-2023---spesialisthelsetjenesten.pdf.

[53] (2025). Forberedt på hardere konkurranse om ansatte [Article in Norwegian]. Helse Sør-Øst.

[54] Norwegian Patient Registry (NPR). The Norwegian Institute of Public Health.

[55] (2024). Norsk diabetesregister for voksne: Data fra diabetespoliklinikker - Diabetes type 1 - Årsrapport for 2023 [Article in Norwegian]. https://www.kvalitetsregistre.no/49e637/siteassets/dokumenter/arsrapporter/diabetesregisteret/arsrapport-2023-norsk-diabetesregister-for-voksne-type-1.pdf.

[56] Chan AW, Tetzlaff JM, Altman DG (2013). SPIRIT 2013 statement: defining standard protocol items for clinical trials. Ann Intern Med.

[57] Norwegian Adult Mental Health Registry (NAMHR). ClinicalTrials.gov.

[58] Norwegian Adult Mental Health Registry (NAMHR). WHO International Clinical Trials Registry Platform (ICTRP).

[59] Bakken IJ, Ariansen AMS, Knudsen GP, Johansen KI, Vollset SE (2020). The Norwegian Patient Registry and the Norwegian Registry for Primary Health Care: Research potential of two nationwide health-care registries. Scand J Public Health.

[60] (2016). Norwegian Registry for Primary Health Care (KPR). The Norwegian Institute of Public Health.

[61] National healthcare quality indicators. Helsedirektoratet.

[62] Healthcare quality and outcomes. Organisation for Economic Co-operation and Development (OECD).

[63] Arah OA, Klazinga NS, Delnoij DMJ, ten Asbroek AHA, Custers T (2003). Conceptual frameworks for health systems performance: a quest for effectiveness, quality, and improvement. Int J Qual Health Care.

[64] Smith PM, Mossialos E, Papanicolas I, Leatherman S (2010). Performance Measurement for Health System Improvement: Experiences, Challenges and Prospects.

[65] ADHD - Nasjonal faglig retningslinje [Article in Norwegian]. Helsedirektoratet.

[66] Selvmordsforebygging i psykisk helsevern og tverrfaglig spesialisert rusbehandling (TSB) - Nasjonal faglig retningslinje [Article in Norwegian]. Helsedirektoratet.

[67] Spiseforstyrrelser - Nasjonal faglig retningslinje [Article in Norwegian]. Helsedirektoratet.

[68] (2017). Nasjonal Faglig Retningslinje Om Bruk Avelektrokonvulsiv Behandling ‐ ECT [Book in Norwegian].

[69] Psykoselidelser – legemiddelbehandling - Nasjonal faglig retningslinje [Article in Norwegian]. Helsedirektoratet.

[70] Evans C, Mellor-Clark J, Margison F, Barkham M, Audin K, Connell J (2000). CORE: clinical outcomes in routine evaluation. J Ment Health.

[71] Üstün TB, Kostanjsek N, Chatterji S, Rehm J (2012). Measuring Health and Disability: Manual for WHO Disability Assessment Schedule (‎WHODAS 20)‎.

[72] Bush K, Kivlahan DR, McDonell MB, Fihn SD, Bradley KA (1998). The AUDIT Alcohol Consumption Questions (AUDIT-C): an effective brief screening test for problem drinking. Arch Intern Med.

[73] Berman AH, Bergman H, Palmstierna T, Schlyter F Drug Use Disorders Identification Test Extended (DUDIT-E). The European Union Drugs Agency (EUDA).

[74] Blampied NM (2022). Reliable change and the reliable change index: still useful after all these years?. tCBT.

[75] Thor J, Lundberg J, Ask J (2007). Application of statistical process control in healthcare improvement: systematic review. Qual Saf Health Care.

[76] Duncan TE, Duncan SC, Strycker LA (2006). An Introduction to Latent Variable Growth Curve Modeling: Concepts, Issues, and Application.

[77] Norwegian Prescribed Drug Registry. The Norwegian Institute of Public Health.

[78] Norwegian Cause of Death Registry. The Norwegian Institute of Public Health.

[79] Norsk helsenett – Cconnecting Norwegian Health Services. Norsk helsenett.

[80] Data protection impact assessment (DPIA). European Data Protection Supervisor.

[81] (2018). Lov om behandling av personopplysninger (personopplysningsloven) [Article in Norwegian]. Lovdata.

[82] (2001). Lov om spesialisthelsetjenesten m.m. (spesialisthelsetjenesteloven) [Article in Norwegian]. Lovdata.

[83] General Data Protection Regulation.

[84] (2019). Regional committees for medical and health research ethics. National Research Ethics Comittees.

[85] Masters R, Anwar E, Collins B, Cookson R, Capewell S (2017). Return on investment of public health interventions: a systematic review. J Epidemiol Community Health.

[86] Gupta P, Gupta M, Koul N (2020). Overdiagnosis and overtreatment; how to deal with too much medicine. J Family Med Prim Care.

